# Hydrogen sulfide protects spinal cord and induces autophagy via miR-30c in a rat model of spinal cord ischemia-reperfusion injury

**DOI:** 10.1186/s12929-015-0135-1

**Published:** 2015-07-07

**Authors:** Lei Li, Hong-kun Jiang, Yun-peng Li, Yan-ping Guo

**Affiliations:** Department of Orthopedics, Shengjing Hospital of China Medical University, Shenyang, 110004 China; Department of Pediatrics, The First Affiliated Hospital of China Medical University, Shenyang, 110001 China

**Keywords:** Autophagy, Beclin-1, miR-30c, Microtubule associated protein 1 light chain 3 (LC3), Oxygen glucose deprivation (OGD)

## Abstract

**Background:**

Hydrogen sulfide (H2S), a novel gaseous mediator, has been recognized as an important neuromodulator and neuroprotective agent in the nervous system. The present study was undertaken to study the effects of exogenous H2S on ischemia/reperfusion (I/R) injury of spinal cord and the underlying mechanisms.

**Methods:**

The effects of exogenous H2S on I/R injury were examined by using assessment of hind motor function, spinal cord infarct zone by Triphenyltetrazolium chloride (TTC) staining. Autophagy was evaluated by expressions of Microtubule associated protein 1 light chain 3 (LC3) and Beclin-1 which were determined by using Quantitative Real-Time PCR and Western blotting, respectively.

**Results:**

Compared to I/R injury groups, H_2_S pretreatment had reduced spinal cord infarct zone, improved hind motor function in rats. Quantitative Real-Time PCR or Western blotting results showed that H2S pretreatment also downregulated miR-30c expression and upregulated Beclin-1 and LC3II expression in spinal cord. In vitro, miR-30c was showed to exert negative effect on Beclin-1 expression by targeting its 3’UTR in SY-SH-5Y cells treated with Oxygen, Glucose Deprivation (OGD). In rat model of I/R injury, pretreatment of pre-miR-30c or 3-MA (an inhibitor for autophagy) can abrogated spinal cord protective effect of H2S.

**Conclusion:**

H2S protects spinal cord and induces autophagy via miR-30c in a rat model of spinal cord hemia-reperfusion injury.

## Background

Ischemia/reperfusion (I/R) injury of the spinal cord is a dynamic process that frequently occurs during a variety of clinical situations such as thoracoabdominal aortic surgery or spinal cord injury [1]. Due to complicated pathogenic factor, such as, membrane permeability variation, fluid and electrolyte disorders, lipid oxidation, it is difficult to achieve effective treatment for spinal cord injury induced by I/R [2].

Hydrogen sulfide (H2S), endogenously derived from L-cysteine in several organs, such as brain, heart, kidney and liver [3], has been regarded as the third gasotransmitter and endogenous neuromodulator and plays multiple roles in the central nervous system under physiological and pathological states, especially in secondary neuronal injury. For example, H2S has been reported to protect brain from I/R injury through maintaining mitochondrial function, inhibiting proinflammatory factors, neutralizing ROS and reducing apoptosis [4]. However, the protective effect of H2S on I/R injury of spinal cord has not been described. Thus, we attempt to investigated the possible regulation role of H2S on spinal cord I/R injury in vitro or in vivo. The treatment with H2S could be achieved by inhalation of H2S or administration of NaHS by intravenous injection. However, the study showed that it is difficult to control the concentration of inhaled H2S, which could result in toxicity to animals [5]. Now, administration of NaHS by intravenous injection becomes the common treatment for I/R injury due to accuracy of concentration [6].

Micro-RNAs (miRNAs) are small noncoding regulatory RNAs of about 19–22 nucleotides that function as translational inhibitor or mRNA degradation promoter by binding to the 3’untranslated region of their target mRNAs [7]. Recently, the biological roles of miRNA has been considered to be extensively involved in the organic I/R injury. For example, Bijkerk et al. miR-126 protect renal ischemia/reperfusion injury by promoting vascular integrity [8]. miR30a/p53 axis plays an important role in triiodothyronine preventing cardiac I/R mitochondrial impairment and cell loss [9]. Although no evidence for miR-30c in spinal cord I/R injury, it has been proved to be a neural protector by targeting brain derived neurotrophic factoras well as neurotrophin signaling and axon guidance [10]. Based on miRNA being involved in H2S protected against ischemic injury [11], we speculates that miR-30c might be involved in H2S protecting I/R injury.

In addition, several miRNAs have also been reported to be involved in autophagy modulation by regulating the expression of autophagy-related genes [12]. Autophagy, a regulated process of degradation and recycling of cellular constituents, can participated in organelle turnover and the bioenergetic management of starvation of spinal cord injury [13]. During autophagy, part of the cytosol or entire organelles are sequestered into autophagosomes. Autophagosomes ultimately fuse with lysosomes, thereby generating single-membraned autophagolysosomes and degrading their content [14]. In experiments, Beclin 1, the mammalian homologue of yeast Atg6, was first described as a Bcl-2-interacting protein , and its mediated autophagy plays an important role in the regulation of cell survival and death [15]. Additionally, microtubule-associated protein 1 light chain 3 (LC3) were previously found to promote autophagy by transforming LC3-I into LC3-II. Notably, it has been reported the early and sustained activation of autophagy after spinal cord injury [16] and the change in expression of these autophagy-related proteins has also been recognized in rat model of spinal cord injury [17]. Recently, autophagy has been reported to contributed to the protective effect of H2S against I/R injury in hepar [18] and colon epithelial cells [19]. Thus, we attempted to testify whether that occurs in regulation effect of H2S on spinal cord I/R injury.

In this study, we established rat model of spinal cord ischemia/reperfusion injury and investigated protective effects of H2S and the underlying mechanism.

## Methods

### Chemicals and reagents

Ham’s F12 medium was obtained from Sigma-Aldrich (St. Louis, USA). Fetal bovine serum (FBS) was from Hyclone (Logan, UT, USA). Lipofectamine 2000 transfection reagent was obtained from Invitrogen Life Technologies (Grand Island, NY, USA). NaHS was purchased from Sigma Aldrich (St. Louis, MO). Antibodies against Beclin-1, LC3-I, LC3-II and β-Actin were purchased from Santa Cruz Biotechnology (Santa Cruz, CA, USA). The Detergent Compatible (DC) Protein Assay kit was purchased from Bio-Rad Laboratories (Hercules, CA, USA).

### Animals and surgery

This study was performed in accordance with the National Institute of Health guidelines for the use of experimental animals, and all animal protocols were approved by the Institutional Animal Care and Use Committee of China Medical University. Male Sprague-Dawley rats (China Medical University Animal Center, Shenyang China) weighting 300 to 350 g were used in the study. The rats were housed in a temperature-controlled environment under a light-dark cycle condition with free access to food and water.

Rat models of spinal cord ischemia-reperfusion injury were performed. Briefly, rats were intraperitoneally anesthetized with 10 % chloral hydrate (300 mg/kg). Rectal temperature was monitored and body temperature was maintained at 37.0 ± 0.5 °C with an infrared heat lamp and a heating pad. A 24-gauge catheter was inserted into the tail artery to monitor the mean distal arterial pressure (MDAP). Spinal cord ischemia was induced by inserting a 2 F-Fogarty balloon catheter (Edwards Life Science, Shanghai, China) through the left femoral artery into the proximal descending thoracic aorta. The distance between the site of insertion to the proximal descending thoracic aorta was approximately 11 cm. The balloon was inflated with 0.05 ml of distilled water to induce spinal cord ischemia. The efficiency of the occlusion was evidenced by an immediate and sustained loss of detectable pulse pressure and a decrease in MDAP.

Twelve hours after ischemia, the balloon was deflated, and spinal cord blood flow was restored. The arterial catheters were then removed, the incisions were sutured. The animals were cultured in appropriate environment for recover. Bladder content was compressed manually as required. Arterial blood samples were collected before ischemia 1 h and 6 h after the beginning of reperfusion, respectively.

### Experimental protocol

The rats were randomly divided into three groups. The sham group (n = 10) underwent the surgical procedure but without aortic occlusion; The I/R injury group (n = 10) underwent the surgical procedure and was administered with an equal volume of saline; The I/R + NaSH group (n = 10) was intraperitoneally injected with NaSH (30 umol/kg, dissolved in saline) 30 minutes before the initiation of ischemia. Forty–eight hours after reperfusion, animals were anesthetized with 3 % halothane and euthanized by transcardiac perfusion with 100 mL of 0.9 % saline solution and 50 mL of 4 % paraformaldehyde in phosphatebuffered saline (PBS).

### Assessment of neurologic function

The hind limb function was scored by the means of the Basso, Beattie, and Bresnahan (BBB) open-field locomotor scale, which ranges from 0 (no detectable hind limb movement) to 21 (normal hind limb locomotion) [20]. Before surgery the rats were placed individually in a molded plastic open field to ensure that all subjects consistently obtained a maximum score of 21. BBB scores were recorded at 1, 6, 12, 24, and 48 hours in the acute phase after reperfusion by five experienced investigators who had not conducted the surgery and were unaware of the group treatments. The final score for each animal was determined by averaging the values from all investigators.

### Staining with triphenyltetrazolium chloride

The ischemic infarct was exhibited by triphenyltetrazolium chloride (TTC) staining and evaluated by computer-aided image acquisition. Briefly, the I/R injured spinal cord were harvested, and sliced into 2.0-mm transverse sections. The slices were incubated in 2 % TTC (dissolved in PBS) for 30 minutes at 37 °C and then were stored in a 10 % formalin solution. Infarct areas were measured by using NIH Image (National Institutes of Health, Bethesda, Md) image analysis software, and data were represented as the percentage of the infarct area in the total area of the spinal cord section.

### Quantitative Real-Time PCR

Total RNA was extracted from the spinal cord tissue or SY-SH-5Y cells with the Trizol Reagent (Invitrogen, Carlsbad, CA, USA), respectively. The mature miR-30(a–e), Beclin-1 mRNA and LC3 miRNA were quantified by using Quantitative Real-Time PCR (Q-RT-PCR) assays with fluorescent nucleic acid dye. Each sample (1 μg) was reverse-transcribed into cDNA by using the RealMasterMix First Strand cDNA Synthesis Kit (Tiangen) according to manufacture’s protocol. Real-time PCR was conducted by using SYBR Premix ExTagTM (Takara) according to the manufacturer’s protocols and performed in the Applied Biosystems 7500 Real-time PCR system. The threshold cycle (CT) is defined as the fractional cycle number at which the fluorescence passes the fixed threshold. The miRNA expression levels were normalized to U6 RNA and the Beclin-1 and LC3 mRNA levels were normalized to actin mRNA. All reactions were run in triplicate.

### Cells and oxygen, glucose deprivation (OGD) treatment

SY-SH-5Y cell line, derived from human hippocampal neurons, was obtained from Sigma-Aldrich (St. Louis, USA) and was cultured in Ham’s F12 medium including EMEM (EBSS) (1:1), 2 mM Glutamine, 1 % Non Essential Amino AcidsNEAA, 15 % Foetal Bovine Serum FBS/FCS at 37 °C. The neuronal injury during ischemia is due to a reduction in the supplement of oxygen and glucose that is named as Oxygen, Glucose Deprivation (OGD). The OGD experiment in vitro is thought to mimic the pathological conditions of ischemia. In the present study, OGD was conducted as described previously [21] in neurons. OGD solution (pH 7.4) containing (in mM): 20 NaHCO3, 120 NaCl, 5.36 KCl, 0.33 Na2HPO4, 0.44 KH2PO4, 1.27 CaCl2, 0.81 MgSO4. Was used. The OGD procedure is just as follows: Neurons were rinsed twice with OGD solution and were incubated in OGD solution in a hypoxic incubator (model 3131, Thermo Forma, San Jose, CA, USA) containing 94 % N2, 1 % O2 and 5 % CO2 for 1 hour; For reoxygenation (Reox), the neurons were incubated in OGD solution containing 5.5 mM glucose at 37 °C in a humidified 5 % CO2 atmosphere for 24 hours. NaSH (10, 100 and 200 μmol/l) was continuously administrated from 1 hour before OGD to the end of the experiment. The same procedure was carried out in the control neurons except the OGD treatment.

### Bioinformatics analysis of miR-545 target in Ku70

Based on bioinformatics analysis, we predicted that hsa-miR-30c can bind with the 3’UTR region of Beclin-1 by using four common websites (Target Scan: http://www.targetscan.org/, miRanda: http://www.microrna.org/, Microcosm: http://www.ebi.ac.uk/enright- srv/microcosm/ cgi-bin/targets/v5/genome.pl, and PITA: http://genie.weizmann.ac.il/) (Fig. 4a).

### Plasmid construction and luciferase reporter assays

For Beclin-1 3’UTR reporter assay, SY-SH-5Y cells were placed in 24-well plates (1 × 10^3^ cells per well) and then transfected with either psi-CHECKTM2-WT-BECN-3’UTR (wild type) or psi- CHECKTM2-MT-BECN-3’UTR that containing the miR-30c targeting sequence (UGUUUAC) (mutant) dual Luciferase reporter plasmid (Promega, WI, USA) according to manufacture’s protocol. The mimics and inhibitors of hsa-miR-30c and their negative controls (RIBO Bio, Guangzhou, P.R. China) were cotransfected with the reporter plasmids at a final concentration of 100 nmol/μl. Forty–eight hours after transfection in neurons, luciferase activity in lysates was measured with a Dual-Luciferase Reporter Assay System (Promega, WI, USA) followed by the manufacture’s suggestions and normalized against the activity of the pRL-SV40. Firefly and Renilla luciferase activities were measured using the Dual-Luciferase Reporter Assay system (Promega, Madison, WI), and the Renilla luciferase activity was normalized to firefly luciferase activity. Independent triplicate experiments were performed for each plasmid construct.

### MTT proliferation assay

SY-SH-5Y cells were seeded at a density of 1000 cells/well. On the next day, after NaSH (100 μmol/l) treatment for 1 hour, cells were treated with OGD. For cell viability assay, 50 mL of 5 mg/mL 3–(4,5–Dimethylthiazol–2-yl) 22,5-diphenyltetrazolium bromide (MTT) was added to wells for 4 h, and then the media were replaced with DMSO for analysis of optical density using a mQuant Microplate Spectrophotometer (BioTek, UK) at a wavelength of 540 nm. All independent analysis were run in triplicate.

### Cell treatment with miR-30c inhibitor or mimics

SY-SH-5Y cells were treated with pre-miR-30c or anti-30c (Ambion Pre-miR miRNA Precursors, Life Technologies) using Oligofectamine (Life Technologies) according to the manufacturer’s instructions. miRNA mimics negative control (pre-NC) and anti-miRNA negative control (anti-NC) were severed as negative controls in the experiments respectively. Further analysis of the samples (infection or RNA isolation) was performed at 48 h post-transfection.

### Western blot

For analysis of protein expression, western blot assay was performed. Briefly, both tissues from the injured spinal cord (extending 2 mm to the infarct, minced with eye scissors) and cells were homogenized in lysis buffer (1 % NP–40, 50 mmol/L Tris, pH 7.5, 5 mmol/L EDTA, 1 % SDS, 1 % sodium deoxycholate, 1 % Triton X–100, 1 mmol/L PMSF, 10 mg/ml aprotinin, and 1 mg/ml leupeptin) and clarified by centrifuging for 20 min in a microcentrifuge at 4 °C. respectively. The total protein concentration was determined with the BCA Protein Assay Kit and the resulting supernatant (50 mg of protein) was subjected to SDS-polyacrylamide gel electrophoresis (PAGE). The separated proteins were transferred to PVDF (Millipore) followed by blocking with 5 % nonfat milk and incubated with primary antibody against Beclin–1 (1:500 ), LC3–I (1:1000), LC3–II (1:1000) or β–actin (1:10000). The target proteins were captured by anti-rabbit horseradish peroxidase-conjugated secondary antibody. Finally, protein was visualized using an enhanced chemiluminescence system (ECL, Pierce Company, USA).

### Statistical analysis

All statistical analyses were performed using SPSS 16.0 software (Chicago, IL). Student’s *t* test was used to analyze the significance between the two groups. One-way Analysis of Variance (ANOVA) was used to test the significance of the Beclin-1 3’UTR. BBB scores data were analyzed by two factor repeated measures analysis of variance (ANOVA; for group and time) followed by post hoc Bonferroni’s test for multiple comparisons. Error bars represent SD. *P*-Values < 0.05 were considered statistically significant.

## Results

### H2S improved I/R-induced spinal cord injury

**Fig. 1:**
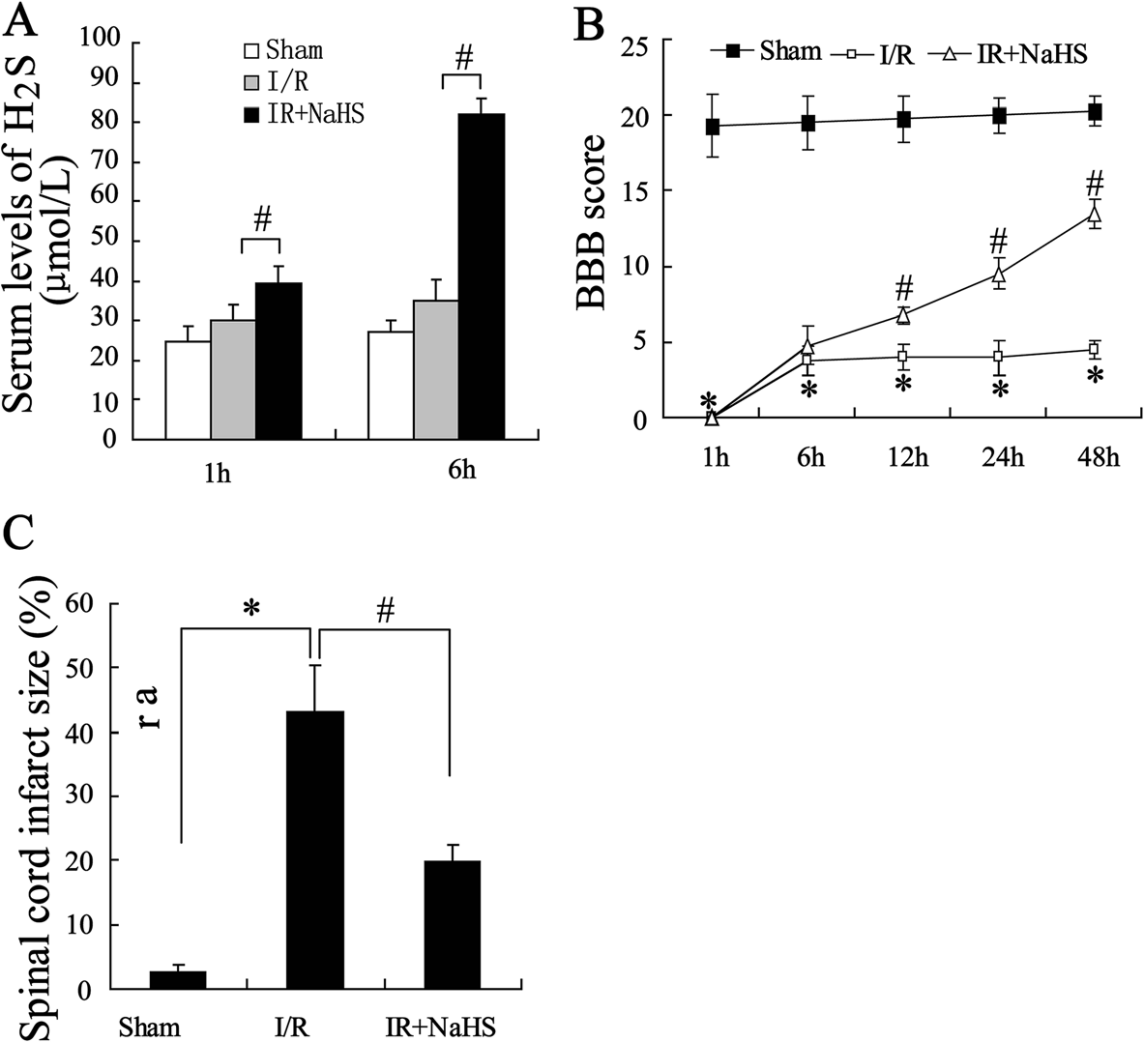
The effect of H_2_S on spinal cord ischemia-reperfusion (I/R) injury in rats. Rats were divided into three groups: sham group, I/R group, treated with NaHS after I/R group. Rat models of spinal cord ischemia-reperfusion injury were established, which were intraperitoneally injected with or without 30 μmol/kg NaHS. **(a)** Serum levels of NaHS were assayed. **(b)** Basso, Beattie, and Bresnahan (BBB) open-field locomotor scale test was performed. **(c)** Quantitative analysis of spinal cord infarction zone. All values are expressed as the mean ± SD. ^*^ *P* < 0.05 vs. sham group; ^#^ *P* < 0.05 vs. I/R group

To identify the effect of H2S on I/R-induced spinal cord injury, exogenous NaSH was intraperitoneal injected into rat models of spinal cord I/R injury 30 minutes prior to the ischemia. The serum level of H_2_S was measured 1 and 6 hours after I/R and the result showed markedly increased the serum concentration of H_2_S 6 h after reperfusion (Fig. 1a). After spinal cord injury, all the rats were paralyzed in both hindlimbs. Moreover, they all had urinary and fecal incontinence. The functional recovery score was followed at five time points between 1 and 48 hours post-I/R injury for both I/R group and NaHS group with the BBB scoring system, a standard method for assessing the hind motor function after SCI in rats. As shown in Fig. 1b, hindlimb locomotor activity improved gradually with increasing time throughout the evaluation period, as the BBB scores gradually increased in both group. Compared to the I/R group, the NaSH group showed significantly improved hindlimb activity score from hour 12 after the injury and on hour 21, the I/R group achieved an average BBB score of 13.8 with consistent plantar stepping and forelimb-hindlimb (FL-HL) coordination. After 48 h of reperfusion, triphenyltetrazolium chloride (TTC) staining was performed to examine infarct zone in spinal cord tissues. Quantitative analysis of spinal cord infarct zone showed that I/R-induced spinal cord injury was obviously improved by NaSH treatment (Fig. 1c).

### H2S promoted autophagy in rat model of spinal cord I/R injury

**Fig. 2:**
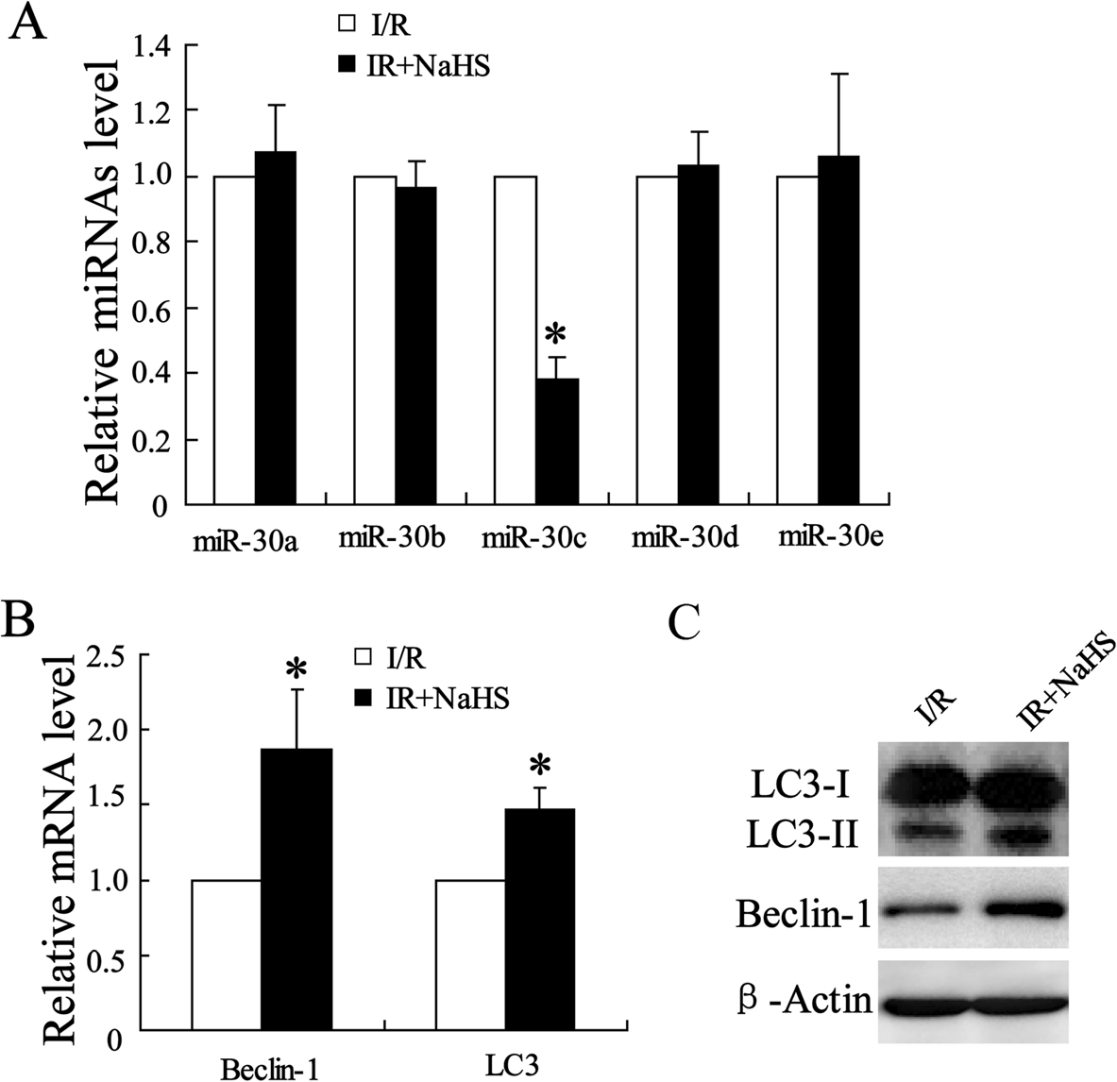
Expression of microRNA30 profiles and autophagy-related protein in rats spinal cord. Spinal cord samples were collected from I/R injury group and its H2S treatment group for molecular detection. **(a)** Quantitative analysis of expression of miR-30 profiles was determined by using Quantitative Real-Time PCR. **(b)** Quantitative analysis of expressions of Beclin-1 mRNA and LC3 mRNA were examined by using Quantitative Real-Time PCR. **(c)** Expression of Beclin-1 and LC3-I/II protein were determined by using western blot assay. All values are expressed as the mean ± SD. ^*^ *P* < 0.05 vs. I/R group

To explore the mechanisms how H_2_S could attenuate I/R-induced spinal cord injury, we examined the expression of miR-30 profiles by using Quantitative Real-Time PCR. The result showed no changes in expression of miR-30 except that decreased in miR-30c (Fig. 2a) suggesting an involvement of miR-30c in neuroprotective effect against spinal cord ischemia-reperfusion injury of H2S. LC3 and Beclin-1 are regarded as the autophagy marker and their levels in spinal cord tissue were also examined. The data showed upregulation in both LC3II and Beclin-1 expression in levels of miRNA and protein (Fig. 2b) suggesting that H_2_S protected spinal cord from I/R injury via increasing cells autophagy.

### H2S improved OGD-induced ischemic injury in SY-SH-5Y cells

**Fig. 3:**
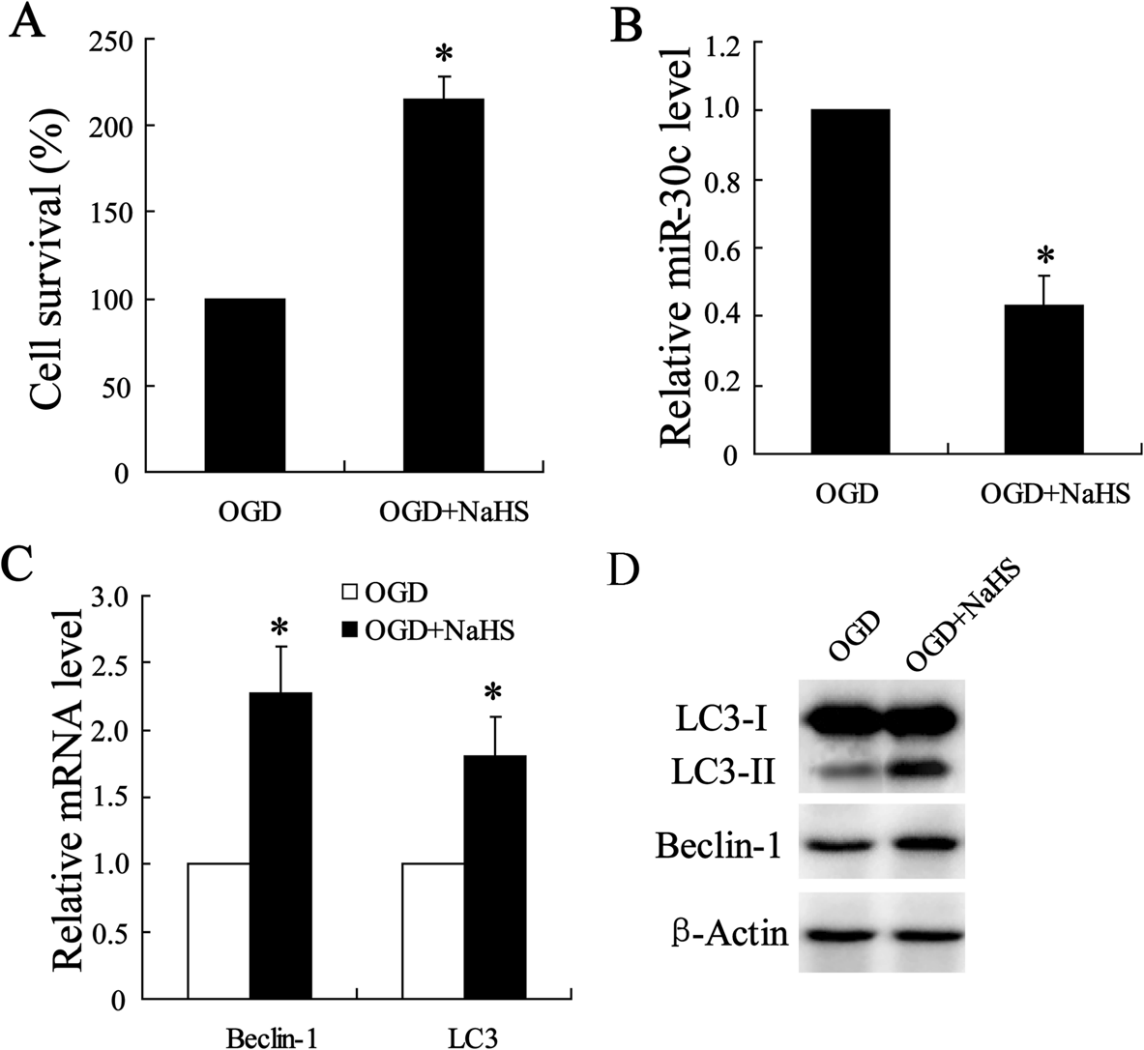
Effect of H_2_S on OGD-induced ischemic injury in SY-SH-5Y cell line. Cell model of OGD injury was established in hippocampal neurons-SY-SH-5Y cell line, which was injected with or without NaHS (100 μmol/l). **(a)** Cell viability was analyzed using MTT assay. **(b)** Quantitative analysis of expression of miR-30 profiles, **(c)** Beclin-1 mRNA and LC3 mRNA were examined by using Quantitative Real-Time PCR. **(d)** Expression of Beclin-1 and LC3-I/II protein were determined by using western blot assay. All values are expressed as the mean ± SD. ^*^ *P* < 0.05 vs. OGD group

To further investigate the mechanisms involved in H2S alleviating I/R-induced spinal cord injury, OGD experiment was performed in hippocampal neurons cell line, SY-SH-5Y. As shown in Fig. 3a, NaSH group showed a significantly increase in cell viability compared with OGD group. Quantitative Real-Time PCR was performed to examined levels of miR-30c expression and the results showed declined expression of miR-30c in cells treated with NaSH (Fig. 3b) which is consistent to that in spinal cord. Accordingly, expressions of Beclin-1 and LC3 were also detected. As shown in (Fig. 3c), both miRNA and protein levels of Beclin-1 and LC3 were significantly enhanced in NaSH group.

### miR-30c acts as a negative regulator of Beclin-1 expression

**Fig. 4:**
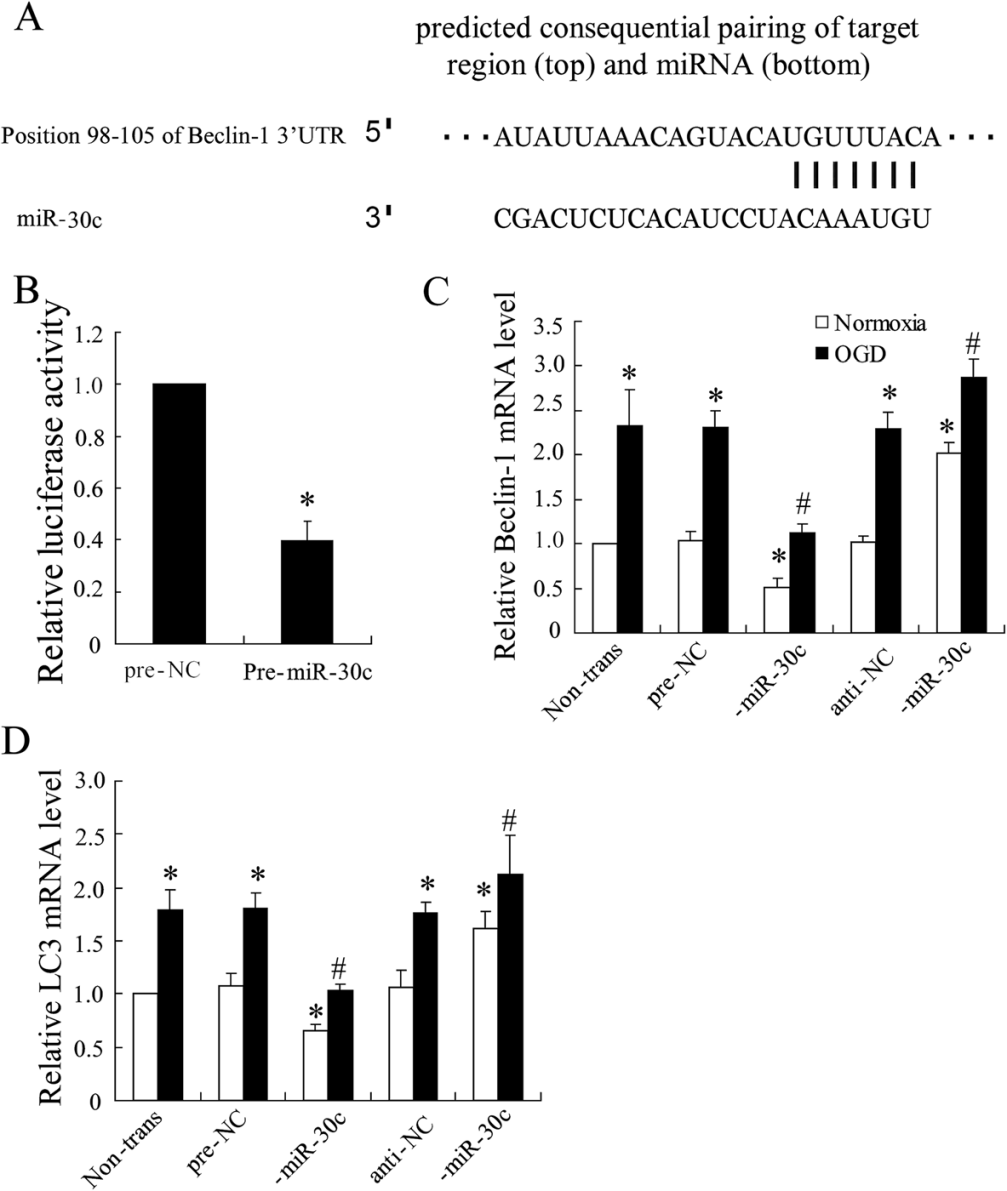
Targeting site of miR-30c in the Beclin-1 3’UTR. **(a)** Beclin-1 3’UTR was predicted a binding site for hsa-miR-30c. The highly conserved mature miR-30c sequence and potential binding between the miR-30c seed region to the mouse Beclin-1 3’UTR sequence are shown. 24 h after transfected with pre-miR-30c or anti-30c, SY-SH-5Y cells were lyzed to analyze **(b)** Beclin-1 3’UTR activity by using luciferase report assay, **(c)** Beclin-1 mRNA and **(d)** LC3 mRNA by using Quantitative Real-Time PCR. All values are expressed as the mean ± SD. ^*^ *P* < 0.05 vs. normoxia negative control group; ^#^ *P* < 0.05 vs. ischemic negative control group

Recent study showed that miR-30 could impair autophagic process by targeting multiple genes in the autophagy pathway [22]. Consistently, in our study of the roles of miR-30 in regulation of autophagy, we observed that only expression of miR-30c showing around 62 % decrease during activation of autophagy under I/R injury conditions in Fig. 2a. Nevertheless, the precise mechanism by which miR-30c affects autophagic process remains unclear. Because the mature sequences are highly conserved between miR-30c and miR-30a, and miR-30a was discovered to act as a negative regulator of autophagy through altering the expression of the key autophagy-promoting gene Beclin-1 [22], we thus tested whether miR-30c could also target Beclin-1 expression. miR-30c is predicted to have the a consensus sequences within the 3’UTR of Beclin-1 (Fig. 4a), we took advantage of the Beclin-1 dual luciferase reporter system to study the role of miR-30c in regulation of Beclin-1 expression. As shown in Fig. 4b, co-transfection of the SY-SH-5Y cells with a pre-miR-30c (100 nM) led to a significant reduction of the reporter gene activity, in comparison with the cotransfection with a control miRNA.

To further demonstrate the effect of miR-30c on Beclin-1 expression, we transfected SY-SH-5Y cells with pre-miR-30c, anti-30c or their negative control, and then examined the expression of Beclin-1 in cells treated with OGD. Fig. 4c showed that treatment with pre-miR-34c suppressed Beclin-1 mRNA expression under ischemia, however, that was increased by cells treated with anti-30c under both ischemia and normoxia. Additionally, we attempted to detected the possible regulated LC3 by miR-30c and the result showed that the LC3 expression varied as Beclin-1 (Fig. 4d) indicating the key regulated role of miR-30c in OGD-induced cells autophagy.

### S_2_H inhibited Beclin-1 3’UTR under OGD

**Fig. 5:**
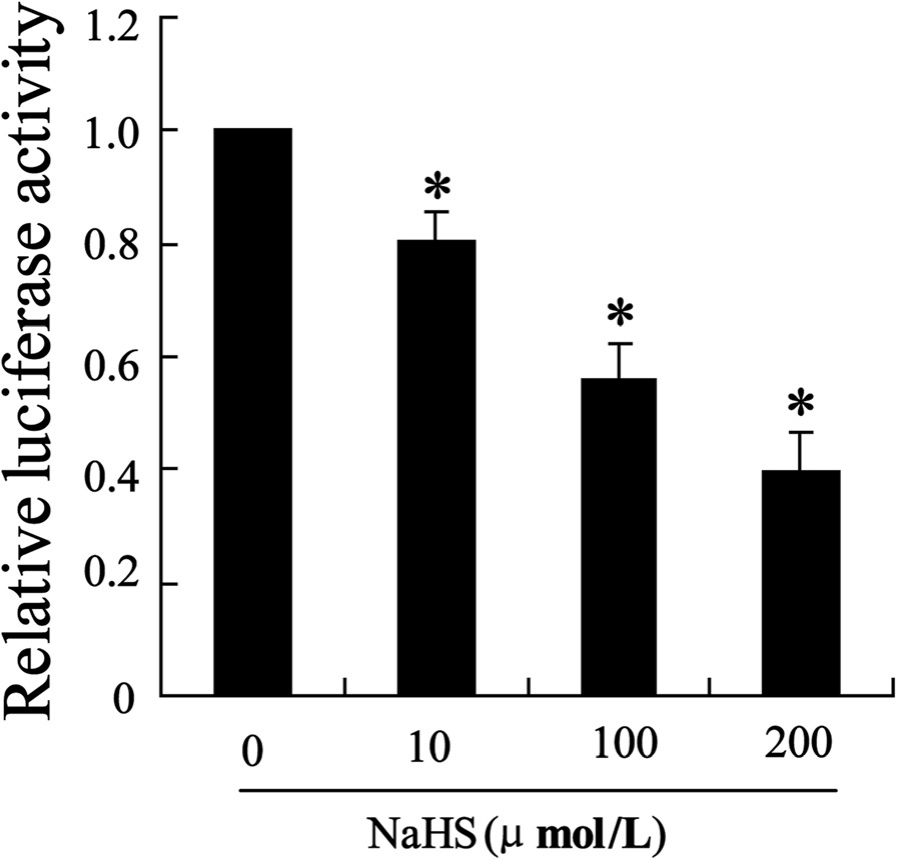
Effect of H_2_S on Beclin-1 3’UTR activity under the condition of ischemic. One hour after SY-SH-5Y cells treated with 10, 100, 200 μmol/l NaHS, cell model of OGD injury was established in hippocampal neurons-SY-SH-5Y cell line. Beclin-1 3’UTR activity was examined by using luciferase report assay. All values are expressed as the mean ± SD. *P < 0.05 vs. cells treated without NaHS

To ascertain the role of miR-30c in protective effect of H2S in autophagy, cells were pretreated NaSH at dose of 10, 100 and 200 μmol/L before OGD treatment and Beclin-1 3’UTR activity was examined. The result showed that NaSH treatment significantly reduced the reporter gene activity in a dose dependent (Fig. 5).

### Spinal core protective effect of H_2_S was reversed by miR-30c overexpression

**Fig. 6:**
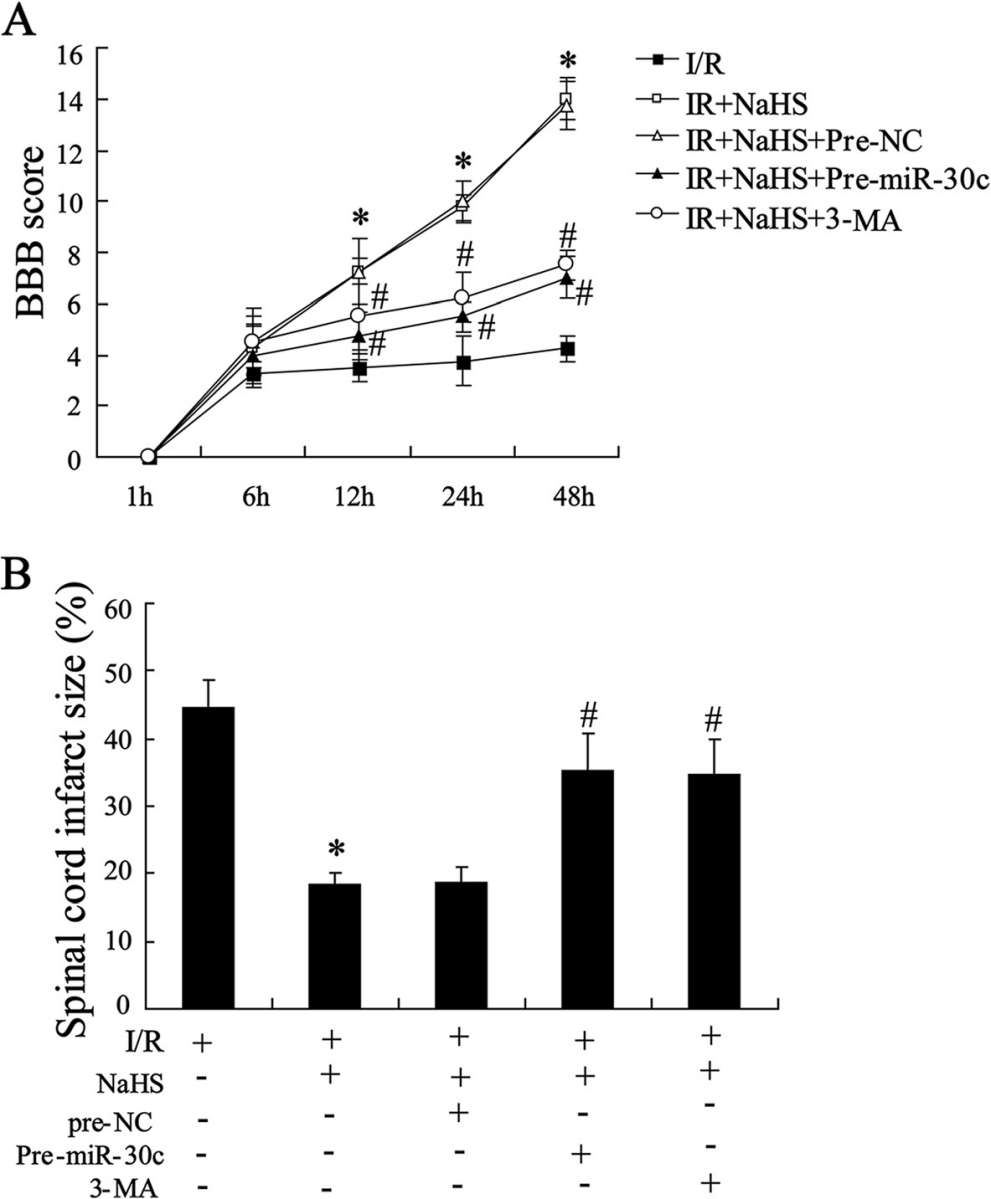
Protective effect of H_2_S on I/R injury was reversed by miR-30c in rats. Rats were pretreated with pre-miR-30c (injected in injury site of the spinal cord 24 h before reperfusion, 100 nmol/l) or 3-MA (intraperitoneally injected, 15 mg/kg) before reperfusion. **(a)** Basso, Beattie, and Bresnahan (BBB) open-field locomotor scale test was performed. **(b)** Quantitative analysis of spinal cord infarction zone. All values are expressed as the mean ± SD. ^*^ *P* < 0.05 vs. I/R group; ^#^ *P* < 0.05 vs. NaSH group

Experiments in SY-SH-5Y cells demonstrated that miR-30c played an important role in H2S-induced autophagy after OGD injury. To assess the effect of miR-30c on the activation of cells autophagy in ischemic spinal cord, BBB scores and infarct zone were detected after reperfusion in rat spinal cord injected with pre-miR-30c or 3-MA, an inhibitor for antophagy. Fig. 6a showed that H2S-induced improvement of hindlimb locomotor activity was reversed by spinal cord treated with pre-miR-30c or 3-MA gradually with increasing time throughout the evaluation period, as the BBB scores gradually decreased in both groups. Additionally, quantitative analysis of spinal cord infarction zone showed that H2S induced-alleviation of I/R spinal cord injury was also obviously abrogated by spinal cord treated with pre-miR-30c or 3-MA (Fig. 6b).

## Discussion

Spinal cord ischemia/reperfusion injury, which occur in secondary neuronal injury after I/R spinal cord injury, is considered as a major health problem that is a frequent cause of disability and death and makes considerable demands on health services. Nevertheless, there was limited advance in the therapeutic strategies to counter spinal cord injury. Thus, neuroprotection and neurorecovery are still the main therapeutic strategies under development. An ideal neuroprotectant would be non-toxic, easily administered, and offer protection at all stages of injury, including prophylaxis. In this study, we exogenously treated spinal cord I/R injury rat with H2S, which acted as a novel signaling molecule in central nervous system [23], by injected with NaSH, and examined the function of spinal cord after injury. Our results showed that NaSH treatment improved the hide motor function and infarct zone in rat model of spinal cord I/R injury. The underlying mechanism is that administration of H2S activates autophagy that indicated by upregulation the expression of autophagy-related proteins including Beclin-1 and LC3II via inhibiting miR-30c expression after spinal cord reperfusion injury.

The time profile of BBB scoring system for hindlimb locomotor function after I/R injury was determined, and time effects of H2S were detected in this study. We observed that pretreatment of NaHS 30 min before I/R injury can alleviate I/R induced spinal cord injury in a manner of time dependent from hour 6 h to hour 48. In fact, previous studies demonstrated that pathological development of I/R results in neuronal cell death [24]. Therefor, after 48 h I/R, measurement of infarct zone was performed in spinal cord tissues. We found in the present study that I/R obviously increased infarct zone that was effectively ameliorated by pretreatment 30 min before I/R injury by NaHS suggesting reducing I/R-induced neural cell lost may be contributed to the neuroprotective effects of H2S. These results was further demonstrated by result that H2S function as a neuroprotective factor for hippocampal neurons that treated by OGD. These findings indicate that H2S may be a potential therapy for spinal cord I/R injury.

Increased autophagy is observed in multiple and distinct experimental models of spinal cord injury [16, 25]. Autophagy plays an important roles in the stability of the body metabolism via affecting the mechanism of apoptosis as well as autophagic cell death. Previous sthudy showed that autophagy is important for the balance between protein synthesis and degradation in spinal cord injury [26]. For example, in cases in which mitochondria are extensively damaged, autophagy may be protective by sequestering and degrading defective mitochondria before they can release death-inducing proteins [27]. Notably, in the early nerve damage, including I/R injury autophagy process is activated to protect nerve cell against injury [28]. This is also observed in our present study. The result of NaSH treatment obviously promoting expression of the LC3II and Beclin-1 which acted as markers for autophagy suggested that H2S protect spinal cord against I/R injury via increased neurocyte autophagy. It was supported by result of inhibition of autophagy by 3-MA abrogated H2S functional outcome after I/R injury in rats.

In addition, we confirmed and are the first to note that the miR-30c was downregulated during spinal cord protective effect of H2S in I/R injury. Previously, several microRNAs have also been reported to be involved in autophagy modulation by regulating the expression of autophagy-related genes [29, 30]. Recent study showed that miR-30 could impair autophagic process by targeting multiple genes in the autophagy pathway [27] . Thus, we hypothesized that there exists the possible targeting region of Beclin-1 mRNA by miR-30c, and it was supported by the result of bioinformatics analysis in Fig. 4a. To confirm this cellular metabolism, hippocampal neurons was transfected with pre-miR-30c or anti-30c to examined expression of Beclin-1. The data indicated the negative regulation of miRNA or protein expression of Beclin-1 by miR-30c.

## Conclusion

The present study demonstrates that systemic administration of H2S improved spinal cord injury and motor function in rat model of I/R injury. H2S may serve as a neuroprotectant to treat I/R-induced spinal cord injury via activating autophagy in a miR-30c dependent signaling pathway. This founding might has potential clinical therapeutic value for treatment of spinal cord injury.
